# The Effect of Domestication and Experience on the Social Interaction of Dogs and Wolves With a Human Companion

**DOI:** 10.3389/fpsyg.2020.00785

**Published:** 2020-04-23

**Authors:** Martina Lazzaroni, Friederike Range, Jessica Backes, Katrin Portele, Katharina Scheck, Sarah Marshall-Pescini

**Affiliations:** ^1^Domestication Lab, Konrad Lorenz Institute of Ethology, University of Veterinary Medicine, Vienna, Austria; ^2^Comparative Cognition, Messerli Research Institute, University of Veterinary Medicine, Vienna, Austria

**Keywords:** free-ranging dogs, wolves, domestication, choice task, hypersociability

## Abstract

The results of current wolf-dog studies on human-directed behaviors seem to suggest that domestication has acted on dogs’ general attitudes and not on specific socio-cognitive skills. A recent hypothesis suggests that domestication may have increased dogs’ overall sociability (hypersociability hypothesis). The aim of the present study was to test one aspect of the hypersociability hypothesis, whereby dogs should be more interested in social human contact compared to wolves, and to investigate the relative roles of both domestication and experience on the value that dogs attribute to human social contact. We compared equally raised wolves and dogs kept at the Wolf Science Center (WSCw, WSCd) but also dogs with different human socialization experiences i.e., pet dogs and free-ranging dogs. We presented subjects with a simple test, divided in two phases: in the Pre-test phase animals were exposed to two people in succession. One person invited the animal for a social/cuddle session (contact provider) and the other fed the animal (food provider). In the Test phase, animals could choose which of the two persons to approach, when both stood in a neutral posture. We directly compared WSCd with WSCw and free-ranging dogs with pet dogs. We found that in the Pre-test, WSCd and free-ranging dogs spent more time with the contact provider than WSCw and pet dogs, respectively. The results regarding the free-ranging dog and pet dog comparison were surprising, hence we conducted a follow-up testing pet dogs in a familiar, distraction-free area. Free-ranging dogs and this group of pet dogs did not differ in the time spent cuddling. In the test phase, WSCd were more likely than WSCw to approach the two experimenters. However, neither for the WSCd-WSCw comparison nor for the free-ranging dogs-pet dogs comparison, we could find a clear preference for one person over the other. Our findings support the idea that domestication has affected dogs’ behavior in terms of their overall interest in being in proximity with a human partner also in case of dogs with a relatively sparse socialization experience (free-ranging dogs). However, it remains unclear what the driving motivation to interact with the human may be.

## Introduction

Based on comparative studies of dogs and wolves (e.g., Frank and Frank, 1982b, 1985; Frank et al., 1989; vonHoldt et al., 2017; Range et al., 2019) a number of hypotheses have been proposed regarding the role that domestication has had in shaping dogs’ cognition, behavior and sociability. Initially, most authors proposed that during domestication humans selected dogs’ social skills to facilitate their cooperative behaviors with them, for example selecting for an increase in their natural attentiveness to humans (Miklósi et al., 2003), or more human-like socio-cognitive abilities (Hare and Tomasello, 2005). These skills and abilities were thus considered novel and unique to dogs. However, more recent studies considering wolves and dogs raised in a similar manner questioned these conclusions, since, when highly socialized with humans, wolves show social abilities (such as cooperating with humans (Range et al., 2019), following human communicative cues (Range and Virányi, 2013; Lampe et al., 2017), and using gazing themselves to communicate (Heberlein et al., 2016) which are similar to those of dogs. These contrasting results sparked a debate regarding the relative role that domestication and experience play in the dog-wolf differences observed, where some authors posited that domestication has a greater effect than life experience in shaping dogs’ human-related socio-cognitive abilities while others suggested that the role of life experience is predominant (e.g., Hare and Tomasello, 2005 vs. Miklósi and Topál, 2005; Riedel et al., 2008 vs. Wynne et al., 2008; Udell et al., 2011 vs. Miklósi and Topál, 2011).

Controlling for life experiences, experiments with similarly raised wolves and dogs nevertheless still show certain differences between the two species. For example, dogs display less aggression and avoidance toward humans than human-socialized wolves (Gácsi et al., 2005) and a higher tendency to seek human social contact (Frank and Frank, 1982b). Moreover, two studies suggested that contrary to wolves (Topál et al., 2005), dogs develop attachment to their owners thereby using them as a ‘safe haven’ in dangerous situations (Gácsi et al., 2013). However, more recent studies showed that wolf puppies also form attachment bonds to humans (Hall et al., 2015; Wheat et al., 2020), although the social bond established between the wolf and the caregiver is not as easily generalizable to other humans as it is in dogs (Gácsi et al., 2005). Furthermore, although dogs and wolves are similarly successful when cooperating with a human in a string-pulling task, wolves are more likely to initiate movement, but less likely to follow the human partners’ initiative than dogs while dogs tend to wait for the human to take the lead (Range et al., 2019).

Taken together, the results of current wolf-dog studies rather support the hypothesis that domestication has acted on dogs’ more general behaviors and not on specific socio-cognitive skills. Domestication may, for example, have reduced dogs’ fearfulness toward humans (Klinghammer and Goodmann, 1987; Scott and Fuller, 2012), decreased the threshold of human social stimulation required for them to be socialized (Miklósi and Topál, 2013), increased their submissive or conflict-avoidance tendencies toward humans (deferential hypothesis, Range et al., 2019) and/or increased their sociability (hypersociability hypothesis, vonHoldt et al., 2017).

In support of the latter hypothesis (hypersociability hypothesis, vonHoldt et al., 2017), researchers presented results showing that when confronted with a human and a puzzle box requiring manipulation to obtain food, pet dogs spend significantly longer engaging in social behavior compared to hand-raised wolves. However, alternative explanations such as wolves’ higher motivation and persistence when confronted with puzzle boxes may suffice to explain such results (Rao et al., 2018). Furthermore, although the wolves in the latter study were human-raised, the comparison with pet dogs remains problematic given that the intensity of human socialization of the two groups are very different (which may in turn affect their human-directed social behavior).

Interestingly, the hypersociability hypothesis posits that the observed genetic changes from wolves to dogs were not specifically selected to create a social bond between dogs and humans. Rather, dogs show genetic predispositions to hypersociality toward any bonded companion. In this regard, experience, in the sense of the degree of socialization with humans (or potentially any other species), still plays a crucial role in determining dogs’ social responses (Udell et al., 2008, 2011; Wynne et al., 2008).

Thus, in order to have a better understanding of the relative roles that domestication and experience play on the expression of sociability toward humans, it would be necessary to adopt two complementary approaches: (1) the comparison of wolves and dogs with the same socialization experience and, (2) the comparison of dog populations with different experiences of human socialization (Miklósi and Topál, 2013). The latter approach has so far rarely been used, with only a few studies including shelter dogs (Udell et al., 2008; Udell, 2015; Brubaker et al., 2019) and free-ranging dogs (Brubaker et al., 2017; Marshall-Pescini et al., 2017a). Interestingly, free-ranging dogs represent almost 80% of the total dog world population (Hughes and Macdonald, 2013; Lord et al., 2013; Pilot et al., 2015) and thus are the main representatives of the dog species deserving much more scientific attention.

Recent studies have shown that, despite their poorer socialization with humans relative to pet-dogs, free-ranging dogs display considerable social skills. For example, they have been shown to be attracted to humans from an early age and to be able to follow the human pointing gesture (Bhattacharjee et al., 2017a) as well as understand human social cues (friendly and threatening), adjusting their behaviors accordingly (Bhattacharjee et al., 2018). Moreover, free-ranging dogs appear to place a high value on social contact with humans. In fact, in a study conducted on Indian free-ranging dogs, the dogs increased their tendency to establish physical contact with an unknown human experimenter after long-term provisioning of a social reward, but not after provisioning of just food (Bhattacharjee et al., 2017b). Taken together these results suggest that free-ranging dogs may also be highly interested in human social contact; however, because in these studies the comparison with pet dogs is lacking, the role of experience in affecting such propensity to human contact cannot be assessed.

The aim of the present study was to further test one aspect of the hypersociability hypothesis, according to which dogs should be more interested in social human contact compared to wolves and, at the same time, to investigate the relative roles of both domestication and experience on the value that dogs attribute to human social contact. Using the complementary approach described above, we compared equally and highly socialized wolves and dogs kept at the Wolf Science Center (Austria) (WSC wolves and WSC dogs) but also dogs with different human socialization experiences i.e., pet dogs and free-ranging dogs. We presented subjects with a simple test, divided in two phases: in the Pre-test phase, animals were exposed to two people in succession. One person invited the animal for a social/cuddle session (contact provider) and the other fed the animal (food provider). In the Test phase, animals could choose which of the two persons to approach, when both stood quietly in a neutral posture.

If the tendency to seek inter-species social proximity has been selected in dogs during the process of domestication (hypersociability hypothesis, vonHoldt et al., 2017), we predict that:

1. In the Pre-test phase, WSC dogs will remain in contact with the contact provider longer than WSC wolves. In the Test phase, WSC dogs will be more likely to approach any of the two experimenters than the wolves and will choose the contact provider more often than WSC wolves.
2. In the Pre-test phase, pet dogs will remain in contact with the contact provider as long as free-ranging dogs. In the Test phase, the two groups will behave similarly: both will approach the experimenters with similar frequencies and choose the contact provider as often as the food provider.

If in dogs the value attributed to the social contact is solely determined by the extent of experience with humans, we predict that:

3. In the Pre-test phase, WSC dogs will remain in contact with the contact provider as long as WSC wolves. In the Test phase, the two groups will behave similarly: both will approach the experimenters with similar frequencies and choose the food provider more often than the contact provider.
4. In the Pre-test phase, pet dogs will remain in contact with the contact provider longer than free-ranging dogs. In the Test phase, pet dogs will be more likely to approach any of the two experimenters than free-ranging dogs and will choose the contact provider more often than free-ranging dogs.

## Materials and Methods

### Ethics Statement

Ethical approval for this study was obtained from the ‘Ethik und Tierschutzkommission’ of the University of Veterinary Medicine of Vienna (Protocol number: ETK-28/07/2017, ETK-05/11/2018, and ETK-022/01/2020). Informed consent was obtained by all owners of the pet dogs. The authorization to test the free-ranging dogs was provided by the municipality of Taghazout (Morocco).

### Subjects

#### Similarly Raised and Kept Wolves and Dogs (WSCw and WSCd)

Similarly raised and kept wolves and dogs (WSCw and WSCd). 16 wolves (6F, 10M; mean age in years: 6.3 ± 3.22 SE) and 13 mixed-breed dogs (6F, 7M: mean age in years: 5.8 ± 1.63 SE) housed at the Wolf Science Center^1^ were tested. All wolves and dogs live in conspecific packs and are raised and kept in the same way. The animals are trained and participate in behavioral tests on a regular basis (for further information on this population) see (Range and Virányi, 2014).

#### Pet Dogs Tested in Dog Areas (PdA)

Mixed-breed pet dogs were tested in outdoor areas in Vienna. Subjects were recruited randomly by asking owners walking around with their dogs if they were willing to participate in the study. A total of 53 pet dogs were tested (22 F; 31 M; mean age in years: 4.34 ± 3.3 SE).

#### Free-Ranging Dogs (FRd)

Free-ranging dogs were tested in their natural environment in the municipality of Taghazout, Agadir, Morocco. The experimenters (ML, LD, KT, and LS) traveled by car to look for solitary dogs (solitary dogs were chosen to avoid interference by conspecifics). Only adult dogs (appearing to be over 1 year of age) were tested. Subjects that appeared uncomfortable with being approached (7 dogs) were not tested. A dog was considered uncomfortable if it showed aggressive behaviors toward the handler (i.e., growling, barking and stiff posture) or an avoidance behavior. A total of 46 dogs were tested (18 F; 28 M). The tested free-ranging dogs were village-dogs living around human settlements and socialized with humans. Despite being socialized with humans and occasionally receiving food by the local people as well as tourists, they are mainly scavengers that feed on garbage and are completely free to move and reproduce.

#### Follow-Up Group: Pet Dogs Tested in a Dog Day Care Facility (PdC)

Following statistical analyses comparing pet dogs tested in dog areas and free-ranging dogs (see section “Results”), an additional group of pet dogs was tested to clarify the obtained results. The group consisted of a total of 31 pet dogs (18 F; 13 M; mean age in years: 4.68 ± 2.94), that regularly frequented an outdoor dog day care facility located in a private garden, which was isolated from possible disturbances. The tests were conducted in this area, which was therefore highly familiar to the dogs.

### Testing Procedure

The procedure varied slightly for the different groups according to the specific environments, where the subjects were tested and the characteristics of the group subjects. All tests consisted of a Pre-test phase and a Test phase. In the Pre-test, the subject received either food or social contact from two different experimenters appearing in sequence. In the Test phase, the subject was free to choose between the two of them. We randomized both the order, in which the subject was exposed to the two experimenters providing food or social contact in the Pre-test phase, as well as the locations in which the two experimenters stood (left or right) in the Test phase.

#### WSCw and WSCd

WSC animals were tested in an outdoor test enclosure at the Wolf Science Center. Before starting the test, the subject was free to explore the enclosure for 10 min. However, if after 10 min the subject was still moving and sniffing around, we gave it more time prior to starting the test.

##### Pre-test

The Food Person (FP) entered the area and stood one meter from the entrance. The subject was in the enclosure free to move and the pre-test started once the subject approached the FP (i.e., the subject looked at the experimenter while approaching her in a 4-m radius). The FP did not call the subject, and once the subject itself approached her, she fed it 5 pieces of dry food (Royal Canin–German Shepherd) within 30 s, by dropping dry food on the ground in front of the subject and avoiding eye contact. After 30 s the FP left the area and hid out of sight of the subject. The subject remained free to move in the enclosure. Then, the Cuddle Person (CP) entered the area and stood one meter from the entrance. Once the subject approached the CP, she made eye contact, squatted down and if the animal came within reach, petted the subject for 30 s speaking nicely with it. After 30 s the CP left the enclosure and hid out of sight of the subject. Then, a third experimenter (henceforth referred to as “handler” and positioned 20 m from the entrance on the opposite side of the testing enclosure) called the subject and fed it with a maximum of 3 pieces of low value dry food (Royal Canin–Medium Adult) once the subject reached her to allow the other persons to enter the test enclosure. The handler was hidden from the subject during the demonstration of the FP and CP. Differently from the test procedure of pet dogs and free-ranging dogs (see below), the handler directed the subject through the fence for safety reasons. Furthermore, to guarantee the animals’ collaboration also in future tests, the subjects were rewarded for coming when called.

##### Test phase

Once the subject was close to the handler and distracted, both the FP and CP re-entered the enclosure without making eye contact with the subject and stood 2 m apart, at a distance of 16 m from the subject. The handler hid once the FP and CP were in the established position. The subject was free to choose to go to either the CP or to the FP. The test started once the subject had seen the experimenters re-entering the enclosure and ended after 1 min. A fourth experimenter, hidden from the subjects’ sight, recorded the trial durations.

Based on previous studies, we have observed that animals at the WSC (in particular wolves) can become uncomfortable when ignored by the trainers (from whom they expect engagement and/or food). Thus, to ensure both trainers and animals were comfortable with the test procedure, the Test phase lasted the maximum duration of one minute, which was enough time to allow animals to make a clear choice. Additionally, it was not possible to conduct the test with WSC animals with unknown people as was done with pet and free-ranging dogs. Therefore, the experimenters were all people who had a close relationship with the animals, such as trainers or hand raisers. For each animal, we chose two trainers that had a similarly close relationship with the subject. However, to take the possible effect of the relationship of the subject with the trainers into account, each subject was tested twice with the same experimenters alternating their roles as FP and CP. However, the order of entrance and the relative positioning of the two experimenters remained stable across the 2 sessions.

WSC animals were fed the day before testing and did not interact with the experimenters acting as FP or CP during the entire day prior to the test being conducted. We kept the same procedure used with WSC animals for pet dogs (PdA and PdC) with the following differences:

1. Pet dogs were tested in an outdoor fenced area in the absence of other dogs. PdA were tested in three different dog areas, while PdC were tested in a familiar fenced area located inside a private garden. All enclosures had different sizes, but in all tests the distances of the experimenters and owners from the entrance of the fenced area were the same.
2. The owner had the role of the handler and was present during the whole test inside the enclosure 6 meters from the entrance used by the FP and CP. The owner was always faced away from the entrance except when calling the subject at the end of the pre-test. Once the subject reached the owner, h/she turned facing the opposite site of the entrance and the FP and CP entered the enclosure.
3. The test phase lasted 2 min.

#### Free-Ranging Dogs (FRd)

We kept the same procedure used with WSC animals with the following differences:

1. Free-ranging dogs were tested in an open environment without restrictions.
2. The three experimenters hid in the car. The pre-test started once the subject recruited by the handler was close to the car. The FP exited from the right side of the car, and after performing the demonstration re-entered the car from the right side. The CP experimenter did the same.
3. Once both FP and CP had performed the demonstration, the handler exited from the left side of the car and led the subject to approximately 5 m from the back of the car, with her back to the car giving the time to the CP and FP to exit from the back of the car (simultaneously). All subjects approached the handler without being called or fed.
4. The test phase lasted 2 min. The handler recorded the trial durations.

All the experimenters and handlers were women. Each session was videotaped with an action camera located above the gate of the main entrance to the enclosure or dog area for WSC animals and pet dogs and on the back of the car for free-ranging dogs. For pet dogs and WSC animals we recorded the test with an additional camera located approximately 5 m to the left or to the right of the action camera.

### Analyses

All the videos were coded using the software Solomon coder (developed by András Péter, Dept. of Ethology, Budapest, www.solomoncoder.com). See Table 1 for definitions of the coded behaviors.

**TABLE 1:** Description of the coded behaviors.

Behavior	Test Phase	Description
Contact	Pre-test	Occurrence (yes/no) and duration of CP touching/stroking the subject.
Choice	Test	The subject touches or approaches to within 20 cm of the experimenter. The first experimenter touched or approached is considered the animal’s choice.
Proximity	Test	The time the subject spends within a half body-length radius of the experimenter.

Inter-observer reliability was carried out between three observers each coding 20% of the video data (Intra-class correlation coefficient: proximity ICC = 0.97; contact ICC = 0.9).

Initial analyses were conducted on WSC animals, free-ranging dogs and pet dogs tested in dog areas (PdA). These analyses were run separately for WSC and non-WSC animals, because of the unavoidable procedural differences; for example, the repeated testing at the WSC required different statistical analyses (see below).

To clarify whether the potentially higher distraction and lower familiarity of dog areas affected pet dog’s behavior, we tested a follow-up group of pet dogs in a dog day care facility (PdC), which dogs frequented regularly. We re-ran all the analyses comparing FrD, PdC and PdA, and report them separately in the results section.

### Analyses of Pre-test Phase: Duration of Contact With the CP

We first tested whether the proportion of time individuals spent in contact with the CP in the pre-test differed between groups (WSCd vs. WSCw, PdA vs. FRd, respectively). To this end we used a Generalized Linear Mixed Model (GLMM) (Baayen, 2008) with beta error structure and logit link function (McCullagh and Nelder, 1989; Bolker, 2008) for the WSCd-WSCw comparison. We included group (dog or wolf) and the side at which the CP was presented as fixed effects and individual ID as a random intercepts effect. For the FRd-PdA comparison we used a Generalized Linear Model (GLM) (Baayen, 2008) with beta error structure and logit link function with the same fixed effects but no random effect as each individual in this data set was tested only once. An identical model was used for the additionally analyses comparing FrD, PdC, and PdA.

### Analyses of the Test Phase

To estimate the extent to which the tested groups (WSCd vs. WSCw or PdA vs. FRd) differed with regard to whether they approached (no or yes) either of the two experimenters (CP, FP) in the test phase, we fitted the same two models (a GLMM for WSCd-WSCd comparison and a GLM for PdA-FRd comparison), but this time with binomial error structure and logit link function (McCullagh and Nelder, 1989) as the response was binary. An identical model was used to additionally compare FrD, PdC, and PdA.

In two further models we addressed the question whether individuals exhibited preferences for one of the two humans based on the choice of the CP or the FP and/or on the amount of time spent in proximity with the CP and FP. With regard to the predictors, these models were identical to those above. In one of these two models the response variable was which of the two humans (CP = 1 or FP = 0) the dog approached, and in the other, the response variable was the proportion of time individuals spent with the CP (out of the total time individuals spent with either of the two experimenters). Hence, the first model was fitted with a binomial error distribution and logit link function and the second with a beta error distribution and logit link function (Bolker, 2008). As before, we fitted both models separately to the WSCd-WSCw data, PdA-FRd data, and subsequently to FrD-PdC-PdA data and again the models for the WSCd-WSCw data were mixed models.

We fitted the models in R (version 3.6.0) (R Core Team, 2019) using the functions glmer of the package lme4 (version 1.1–21; Bates et al., 2014; GLMM with binomial error distribution), glmmTMB of the identically named package (version 0.2.3; Brooks et al., 2017; GLMM with beta error distribution), glm of the R stats package (GLM with binomial error distribution), or betareg of the identically named package (Zeileis et al., 2010) (version 3.1–2; GLM with beta error distribution). We determined model stability by excluding individuals one at a time and comparing the estimates derived for these subsets of data with those obtained for the full data set. The fitted models appeared to be of moderate to good stability (for details see the results section). In the case of GLMMs, we determined confidence intervals of the estimated coefficients and the fitted model by means of parametric bootstraps (N = 1000; functions bootMer of the package lme4 or simulat, e.g., lmmTMB of the package glmmTMB). For the beta GLM, we determined confidence intervals of model estimates using the R function confint and confidence intervals of the fitted model by means of a non-parametric bootstrap (N = 1000). In the case of GLMMs we determined the significance of individual effects using likelihood ratio tests (Dobson, 2002), comparing the fit of the respective full model with that of reduced models lacking the fixed effects one at a time (Barr, 2013), otherwise we used Wald’s z-approximation (Field, 2005). None of the models with beta error distribution were overdispersed (dispersion parameters: comparison WSCd-WSCw, contact model: 0.688 proximity model: 1.045; comparison PdA-FRd, contact model: 1.083; proximity model: 0.976; comparison FrD-PdC-PdA, contact model: 0.991; proximity model: 1.127). For sample sizes and the number of choices of the two experimenters in the case of logistic models, see the Supplementary Material and Table 1.

## Results

### WSCd-WSCw Comparison

#### Pre-test Phase

**FIGURE 1:**
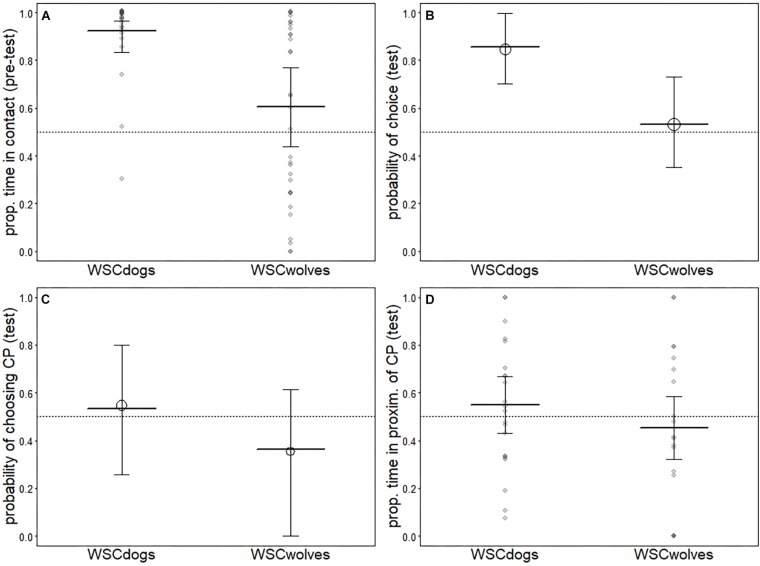
Results for the models comparing WSC dogs and WSC wolves. Indicated are the average response [circles in **(B)** and **(C)**] as well as the fitted model (thick horizontal lines) and its confidence intervals (error bars). Darker dots in **(A)** and **(D)** depict observations falling on top of one another.

In the pre-test phase, all dogs in both tests accepted being cuddled by the trainer. Despite all wolves approached the trainer, then two subjects did not accept the contact in one of the two tests performed (one subject in the first test and one subject in the second test). There was a clear difference between WSC dogs and WSC wolves (see Table 2) whereby most dogs spent large fractions of their time in contact with the CP whereas wolves, on average, spent only about half the proportion of their time in contact with the CP. However, variation among wolves was large (WSCd, first test: mean = 28.09, dev.st = 5.95; second test: mean 28.72, dev.st = 4.52. WSCw, first test: mean = 19.28, dev.st 11.69; second test: mean = 17.83, dev.st = 12.28) (see Figure 1A).

**TABLE 2:** Results of the WSCd-WSCw comparison regarding the time spent in contact with the CP in the pre-test phase (estimates, together with standard errors, confidence limits, tests, as well as minimum and maximum of estimates derived after excluding individuals one at a time).

Term	Estimate	SE	Lower Cl	Upper Cl	χ^2^	*df*	*P*	Min	Max
Intercept	2.512	0.464	1.567	3.335			^(1)^	2.413	2.832
Group^(2)^	−2.088	0.594	−3.172	−0.769	10.783	1	0.001	−2.349	−1.858

*^(1)^not indicated because of having a very limited interpretation. ^(2)^dummy coded with WSC dogs being the reference category.*

#### Test Phase

Overall, we found that WSC dogs were more likely to make a choice than WSC wolves (see Table 3 and Figure 1B). In the first test, one WSC dog did not make a choice, whereas seven chose the FP and five chose the CP (exact binomial test: pet dogs *p* = 0.38); six WSC wolves did not make a choice (just ignoring the people and sniffing/walking around); six individuals chose the FP and four individuals chose the CP (exact binomial test: *p* = 0.37). In the second test, three WSC dogs did not make a choice, whereas three chose the FP and seven chose the CP (exact binomial test: pet dogs *p* = 0.17); nine WSC wolves did not make a choice, five individuals chose the FP and two individuals chose the CP (exact binomial test: *p* = 0.22). Thus, both groups did not show a significant preference for either person. Wolves and dogs did not differ in the probability to approach the CP (see Table 4 and Figure 1C) nor in the proportion of time spent in her proximity (time in proximity with CP: WSCd, first test: mean = 4.33, dev.st = 5.76; second test: mean = 4.41, dev.st = 5.81. WSCw, first test: mean = 4.46, dev.st 5.69; second test: mean = 4.35, dev.st = 5.65. Time in proximity with FP: WSCd, first test: mean = 5.74, dev.st = 9.22; second test: mean = 5.77, dev.st = 9.22. WSCw, first test: mean = 5.85, dev.st 9.01; second test: mean = 5.79, dev.st = 9.04) (see Table 5 and Figure 1D).

**TABLE 3:** Results of the WSCd-WSCw comparison regarding the willingness to approach the experimenters (estimates, together with standard errors, confidence limits, tests, as well as minimum and maximum of estimates derived after excluding individuals one at a time).

Term	Estimate	SE	Lower Cl	Upper Cl	χ^2^	*df*	*P*	Min	Max
Intercept	1.545	0.669	0.583	4.961			^(1)^	1.442	2.163
Group^(2)^	–1.659	0.736	–4.490	–0.615	6.679	1	0.009	–2.329	–1.473
Social^(3)^	0.490	0.614	–0.709	2.176	0.650	1	0.419	0.325	0.767

*^(1)^not indicated because of having a very limited interpretation. ^(2)^dummy coded with WSC dogs being the reference category. ^(3)^side at which the CP was placed; dummy coded with left being the reference category.*

**TABLE 4:** Results of the WSCd-WSCw comparison regarding the approach to the CP experimenter (estimates, together with standard errors, confidence limits, tests, as well as minimum and maximum of estimates derived after excluding individuals one at a time).

Term	Estimate	SE	Lower Cl	Lpper Cl	χ^2^	*df*	*P*	Min	Max
Intercept	0.585	0.540	–0.467	11.449			^(1)^	0.421	0.834
Group^(2)^	–0.704	0.680	–20.487	0.665	1.090	1	0.296	–0.932	–0.478
Social^(3)^	–0.871	0.670	–21.567	0.609	1.719	1	0.189	–1.162	–0.676

*^(1)^not indicated because of having a very limited interpretation. ^(2)^dummy coded with WSC dogs being the reference category. ^(3)^side at which the CP was placed; dummy coded with left being the reference category.*

**TABLE 5:** Results of the WSCd-WSCw comparison regarding the proportion of time spent in proximity of the CP (estimates, together with standard errors, confidence limits, tests, as well as minimum and maximum of estimates derived after excluding individuals one at a time).

Term	Estimate	SE	Lower Cl	Upper Cl	χ^2^	*df*	*P*	Min	Max
Intercept	0.484	0.299	–0.055	1.107			^(1)^	0.381	0.610
Group^(2)^	–0.385	0.355	–1.124	0.274	1.162	1	0.281	–0.561	–0.266
Social^(3)^	–0.595	0.357	–1.374	0.072	2.708	1	0.099	–0.820	–0.441

*^(1)^not indicated because of having a very limited interpretation. ^(2)^dummy coded with WSC dogs being the reference category. ^(3)^side at which the CP was placed; dummy coded with left being the reference category.*

### Free-Ranging Dogs-Pet Dogs (PdA) Comparison

#### Pre-test Phase

Although all dogs in both groups approached the experimenter in the pre-test, 90.56% (48 of 53) of pet dogs (tested in dog areas, PdA) and 86% (39 of 45) of free-ranging dogs accepted cuddling. PdA spent less time in contact with the CP than free-ranging dogs (PdA: mean = 15.28, dev.st = 11.37; free-ranging dogs: mean = 24.43, dev.st = 11.32) (see Table 6).

**TABLE 6:** Results of the FRd-PdA comparison regarding the duration of time spent in contact with the CP in the pre- test (estimates, together with standard errors, tests, confidence limits, as well as minimum and maximum of estimates derived after excluding individuals one at a time).

Term	Estimate	SE	*z*	*P*	Lower Cl	Upper Cl	Min	Max
Intercept	0.721	0.188		^(1)^	0.351	1.091	0.684	0.820
Group^(2)^	–0.923	0.253	-3.639	< 0.001	–1.419	−0.426	–1.030	–0.886

*^(1)^not indicated because of having a very limited interpretation. ^(2)^dummy coded with free-ranging dogs being the reference category.*

#### Test-Phase

Both pet dogs (tested in dog areas, PdA) and free-ranging dogs did not show a significant preference for the CP or the FP (see Tables 7–9). Fifteen PdA did not make a choice, whereas 21 chose the FP and 17 chose the CP (exact binomial test: *p* = 0.31). Fourteen free-ranging dogs did not make a choice, 20 individuals chose the FP and 12 individuals chose the CP (exact binomial test: *p* = 0.10). The two groups did not differ in the probability to approach the CP nor in the proportion of time spent in her proximity (time in proximity with CP: PdA mean = 5.39, dev.st = 17.62; FrD: mean = 14.79, dev.st = 28.47; time in proximity with FP: PdA mean = 3.99, dev.st = 10.75; FrD: mean = 7.9, dev.st = 16.95) (see Tables 7–9).

**TABLE 7:** Results of the FRd-PdA comparison regarding the willingness to approach the experimenters (estimates, together with standard errors, tests, confidence limits, as well as minimum and maximum of estimates derived after excluding individuals one at a time).

Term	Estimate	SE	*z*	*P*	Lower Cl	Upper Cl	Min	Max
Intercept	1.240	0.434		^(1)^	0.431	2.152	1.099	1.298
Group^(2)^	0.179	0.451	0.396	0.692	–0.710	1.069	0.101	0.256
Social^(3)^	–0.780	0.469	–1.662	0.097	–1.738	0.118	–0.840	–0.671

*^(1)^not indicated because of having a very limited interpretation. ^(2)^dummy coded with free-ranging dogs being the reference category. ^(3)^side at which the CP was placed; dummy coded with left being the reference category.*

**TABLE 8:** Results of the FRd-PdA comparison regarding the approach to the CP (estimates, together with standard errors, tests, confidence limits, as well as minimum and maximum of estimates derived after excluding individuals one at a time).

Term	Estimate	SE	*z*	*P*	Lower Cl	Upper Cl	Min	Max
Intercept	–0.488	0.439		^(1)^	–1.381	0.360	–0.584	–0.352
Group^(^^2)^	0.246	0.493	0.499	0.618	–0.718	1.225	0.147	0.320
Social^(3)^	0.059	0.489	0.120	0.904	–0.904	1.024	–0.025	0.145

*^(1)^not indicated because of having a very limited interpretation. ^(2)^dummy coded with free-ranging dogs being the reference category. ^(3)^side at which the CP was placed; dummy coded with left being the reference category.*

**TABLE 9:** Results of the FRd-PdA comparison the proportion of time spent in proximity of the CP (estimates, together with standard errors, tests, confidence limits, as well as minimum and maximum of estimates derived after excluding individuals one at a time).

Term	Estimate	SE	*z*	*P*	Lower Cl	Upper Cl	Min	Max
Intercept	0.222	0.279		^(1)^	−0.324	0.769	0.166	0.303
Group^(2)^	0.079	0.342	0.232	0.817	−0.590	0.749	0.020	0.148
Social^(3)^	–0.385	0.342	–1.125	0.260	−1.056	0.286	–0.450	–0.337

*^(1)^not indicated because of having a very limited interpretation. ^(2)^dummy coded with free ranging-dogs being the reference category. ^(3)^side at which the CP was placed; dummy coded with left being the reference category.*

### Additional Analyses: PdA-PdC-PdA Comparison

#### Pre-test Phase

**FIGURE 2:**
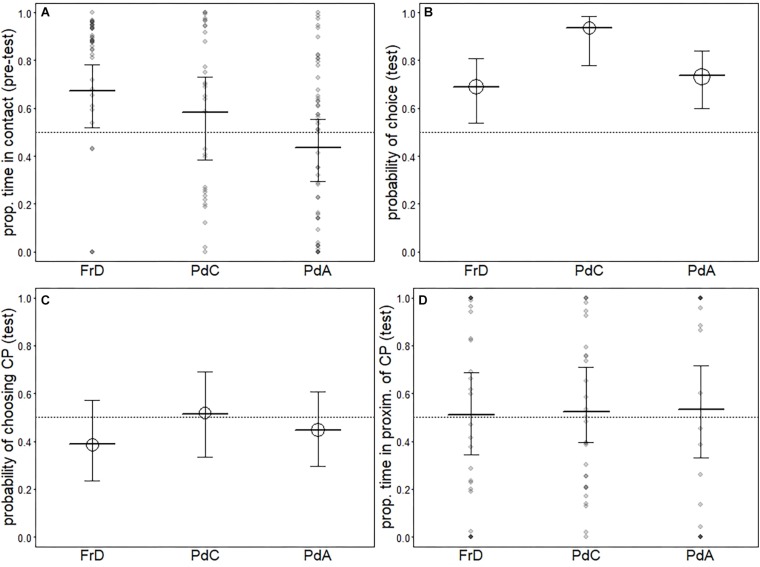
Results for the models comparing free-ranging dogs (FrD), pet dogs tested in the dog care facility (PdC) and pet dogs tested in dog areas (PdA). Indicated are the average response [circles in **(B)** and **(C)**] as well as the fitted model (thick horizontal lines) and its confidence intervals (error bars). Darker dots in **(A)** and **(D)** depict observations falling on top of one another.

In the pre-test phase, we found that pet dogs tested in dog areas (PdA) spent less time in contact with the CP than free-ranging dogs (FrD) and pet dogs tested in the dog care facility (PdC), and no difference was found between FrD and PdC (PdC: mean = 18.05, dev.st 10.30) (see Table 10 and Figure 2A).

**TABLE 10:** Results of the FrD-PdC-PdA comparison regarding the duration of time spent in contact with the CP in the pre- test (estimates, together with standard errors, tests, confidence limits, as well as minimum and maximum of estimates derived after excluding individuals one at a time).

Term	Estimate	SE	*z*	*P*	Lower Cl	Upper Cl	Min	Max
Intercept	0.715	0.187		^(1)^	0.348	1.083	0.675	0.811
Group (PdC)^(2)^	–0.379	0.283	–1.339	0.180	–0.934	0.176	–0.464	–0.286
Group (PdA)^(2)^	–0.980	0.254	–3.860	<0.001	–1.478	–0.482	–1.084	–0.941

*^(1)^not indicated because of having a very limited interpretation. ^(2)^dummy coded with FrD being the reference category; the difference between PdC and PdA was estimated as 0.601 ± 0.274, *z* = –2.189, *P* = 0.028.*

#### Test Phase

We found that pet dogs tested in the day care facility (PdC) were more likely to make a choice than pet dogs tested in dog areas (PdA) and free-ranging dogs (FrD) (see Table 11 and Figure 2B). Two PdC did not make a choice, whereas 14 individuals chose the FP and 15 individuals chose the CP (exact binomial test: *p* = 0.48). Thus, as all other groups, PdC did not show a significant preference for either person. All groups did not differ in the probability to approach the CP (see Table 12 and Figure 2C) nor in the proportion of time spent in her proximity (time in proximity with CP: mean = 16.73, dev.st = 23.81; time in proximity with FP: mean = 12.15, dev.st = 14.18) (see Table 13 and Figure 2D).

**TABLE 11:** Results of the FrD-PdC-PdA comparison regarding the willingness to approach the experimenters (estimates, together with standard errors, tests, confidence limits, as well as minimum and maximum of estimates derived after excluding individuals one at a time).

Term	Estimate	SE	z	*P*	Lower Cl	Upper Cl	Min	Max
Intercept	1.160	0.421		^(1)^	0.371	2.039	1.027	1.217
Group (PdC)^(2)^	1.913	0.803	2.382	0.017	0.520	3.825	1.573	1.992
Group (PdA)^(2)^	0.236	0.454	0.519	0.604	–0.657	1.134	0.157	0.313
Social^(3)^	–0.647	0.449	–1.440	0.150	–1.559	0.215	–0.704	–0.549

*^(1)^not indicated because of having a very limited interpretation. ^(2)^dummy coded with FrD being the reference category; the difference between PdC and PdA was estimated as 1.677 ± 0.798, *z* = –2.101, *P* = 0.035. ^(3)^side at which the CP was placed; dummy coded with left being the reference category.*

**TABLE 12:** Results of the FrD-PdC-PdA comparison regarding the approach to the CP (estimates, together with standard errors, tests, confidence limits, as well as minimum and maximum of estimates derived after excluding individuals one at a time).

Term	Estimate	SE	*z*	*P*	Lower Cl	Upper Cl	Min	Max
Intercept	–0.623	0.423		^(1)^	–1.485	0.191	–0.705	–0.493
Group (PdC)^(2)^	0.509	0.526	0.969	0.332	–0.516	1.556	0.414	0.602
Group (PdA)^(2)^	0.236	0.494	0.477	0.633	–0.730	1.217	0.139	0.312
Social^(3)^	0.331	0.411	0.805	0.420	–0.472	1.143	0.269	0.387

*^(1)^not indicated because of having a very limited interpretation. ^(2)^dummy coded with FrD being the reference category; the difference between PdC and PdA was estimated as 0.274 ± 0.496, *z* = –0.552, *P* = 0.581. ^(3)^side at which the CP was placed; dummy coded with left being the reference category.*

**TABLE 13:** Results of the FrD-PdC_PdA comparison the proportion of time spent in proximity of the CP (estimates, together with standard errors, tests, confidence limits, as well as minimum and maximum of estimates derived after excluding individuals one at a time).

Term	Estimate	SE	*z*	*P*	Lower Cl	Upper Cl	Min	Max
Intercept	0.072	0.263		^(1)^	–0.442	0.583	0.003	0.144
Group (PdC)^(2)^	0.053	0.336	0.157	0.875	–0.607	0.702	–0.009	0.119
Group (PdA)^(2)^	0.093	0.342	0.272	0.786	–0.576	0.756	0.029	0.163
Social^(3)^	–0.061	0.280	–0.218	0.827	–0.600	0.492	–0.102	–0.009

*^(1)^not indicated because of having a very limited interpretation. ^(2)^dummy coded with FrD being the reference category; the difference between PdC and PdA was estimated as 0.040 ± 0.355, *z* = 0.113, *P* = 0.910. ^(3)^side at which the CP was placed; dummy coded with left being the reference category.*

## Discussion

Overall, we found that human-socialized wolves seemed to be less attracted to humans (despite being closely bonded individuals) than similarly raised dogs, highlighting the important role of domestication in affecting dogs’ social behaviors toward humans. Moreover, the results obtained from the comparison between highly socialized pet dogs and free-ranging dogs seem to suggest that even a limited/reduced socialization experience with humans is sufficient to elicit a strong social response in dogs. However, the subjects’ motivation for interacting with humans remain unclear.

We found that while WSC dogs spent almost the entire duration of the Pre-test social phase being cuddled by the experimenter (medium 91.81%, range 30.43% – 100%, dev. Stand 16.27%), wolves spent only half of their available time in contact with the person (medium 57.76%, range 0% – 100%, dev. Stand 36.24%), although the variability in the response was much larger in wolves than dogs. Given that subjects were free to move and were never called by their names, we assume that their behaviors were not influenced by the test setting and/or their responsiveness to being asked to do something, but rather reflect their interest in socially interacting with the human or exploring the environment. When a subject was not in contact with the experimenter, it was typically moving around exploring the enclosure, thus the more explorative attitude of wolves compared to dogs (Moretti et al., 2015, Marshall-Pescini et al., 2017b) might have taken precedence over their proximity-seeking toward the experimenter. However, contrary to wolves, dogs were attracted to the presence of the human, which for them seemed to be the most interesting stimulus in the environment. In any case, it is important to note that the experimenters were hand raisers, with whom both the wolves and the dogs have had a close bond. This might have increased wolves confidence in interacting with them since, as previously observed, wolves are less likely than dogs to generalize their social response to unknown humans (Gácsi et al., 2005), or on the contrary, might have decreased wolves’ interest in the experimenters due to the lack of novelty.

Similarly, we found a significant difference between dogs and wolves in the likelihood of approaching either one of the two experimenters at the beginning of the Test-phase (92.3% of dogs approached in the first test and 77% in the second, 62.5% of wolves approached in the first test and 43.7% in the second). Thus, as well as having a greater tendency than wolves to accept the social contact of an ‘active’ human (Pre-test), dogs were also more likely to actively seek out human proximity than wolves when the two experimenters re-appearing in the Test phase maintained a neutral posture and completely ignored them. However, it is interesting to note that in the second test, in which animals already experience that the two people would not be doing much with them, we found a similar decrease in the number of subjects approaching the experimenters in both wolves and dogs.

Our results are in line with the findings of a previous study measuring sociability of pet dogs and captive hand-raised wolves, in which dogs spent more time in proximity to a human than wolves both when the human was ‘active’ (calling and touching the subject) and when s/he was ignoring the subject (Bentosela et al., 2016). Despite the authors acknowledging that differences in the experience of the two groups might have affected the results, in light of our own results on similarly raised groups, it seems that the experience with humans might have only modestly affected the differences observed between wolves and dogs regarding these behaviors. Considering these results, a more in depth investigation on differences in sociability between dogs and wolves is currently in progress at the Wolf Science Center on similarly raised populations.

Despite wolves showing less attraction to humans than dogs overall, the variance in the time spent accepting the social contact was large – from 0% to 100% – (see similar results also in Bentosela et al. (2016) with pet dogs vs. hand-raised wolves. The wolves’ variability is likely to be the basis on which selection has acted during the domestication process (Persson et al., 2017) and the smaller variance observed in dogs compared to wolves supports the idea that dogs have undergone a strong selective process for higher sociability. Interestingly, we also found a wide variance in the time spent accepting the social contact in pet dogs tested in dog areas, suggesting that the role of life experience might be of great importance in affecting subjects’ sociability.

As pointed out by Miklósi and Topál (2013), due to an effect of the major evolutionary processes, species may differ (be constrained) in the degree to which they are able to react to challenges of the social environment showing a difference in their phenotypic plasticity. Thus, due to domestication, dogs may require a lower intensity of social stimulation than wolves to display a similar social response toward human, but still life experience plays an important role in affecting subjects’ behavior.

Given the previous results, we would have expected WSC dogs to choose more often the ‘social experimenter’ than WSC wolves, however, this was not the case, and additionally, also within species, no differences in their preferences emerged (overall 10 of 26 times dogs chose the FP and 12 of 26 the CP, while 11 of 32 times wolves chose the FP and 6 of 32 chose the CP). If dogs were more likely to approach than wolves because of a higher social or food motivation, in the test phase, we would have found a clear preference for either the ‘social experimenter’ or the ‘food experimenter,’ respectively. Thus, the absence of a clear preference for one experimenter does not allow us to draw any conclusion regarding the subjects’ motivation for approaching the humans. It is possible that subjects did not remember, which of the two experimenters provided food or social contact in the pre-test phase. Another possibility is that, since these animals are used to interacting with the trainers and receiving both food and social contact from them, it might be that they did not make a clear distinction between the possibilities of receiving food or social contact when approaching a trainer. This interpretation is supported by the findings that subjects were not consistent in their choice in the two tests overall. In fact, only four dogs and one wolf were consistent in choosing either food or social contact in both tests (2 dogs chose social contact and 2 dogs chose food; the wolf chose food). However, nine animals (6 of 13 dogs and 3 of 16 wolves) were consistent in their choice of the trainers (regardless of their role) across the tests. Thus, the possibility that the relationship with the trainer was affecting the subject’s choice cannot be excluded.

Some of our findings relating to the wolf-dog comparison are in line with one aspect of the hypersociability hypothesis, which posits that the process of domestication has resulted in dogs showing a hypersocial response toward humans (and other species- although this aspect has not, as of yet, been tested) (vonHoldt et al., 2017). However, given that subjects’ motivation for approaching the humans remains unclear, we suggest that other factors should also be taken into account, when explaining the wolf-dog differences in human-directed sociability. In fact, dogs’ behaviors might have been determined by their more deferential attitude toward humans compared to wolves (Deferential Behavior Hypothesis, Range et al., 2019). Thus, dogs would have a greater acceptance of contact with humans compared to wolves, as well as being more prone to approach the human, considered to be a leader. Indeed this interpretation is in line with observational studies showing that greeting behavior is most often observed between conspecifics from the subordinate to the most dominant individuals in both wolf and dog packs (Cafazzo et al., 2010, 2016). Finally, the more explorative attitude of wolves compared to dogs might have additionally played a role in diverting wolves’ attention to other aspects of their environment.

We found that despite the majority of both pet dogs and free-ranging dogs accepted the experimenter’s cuddle session in the Pre-test social phase, free-ranging dogs spent a greater amount of the available time being cuddled than pet dogs tested in dog areas (free-ranging dogs: mean = 24.43; pet dogs: mean = 15.28). This finding contrasts with our predictions.

One possible reason for these unexpected results is a bias in our selection of animals. While we tested all pet dogs whose owners agreed to do the tests, we were potentially more biased when selecting free-ranging dogs. This might have affected the difference observed between the two groups. However, when selecting free-ranging dogs, we only excluded subjects that showed aggressive or extreme avoidance behaviors when initially approached by the handler (N = 7). Potentially dog owners, who did not participate in the test when asked by the experimenters, made a similar selection, refusing to participate in the study if they knew their dogs to be aggressive or fearful toward strangers (although this cannot be confirmed). The overall longer acceptance of social contact by free-ranging dogs compared to pet dogs tested in the dog areas might be due to the former’s greater desire for contact potentially driven by the free ranging-dogs’ lack of human social contact, as has been already suggested for shelter dogs. For example, in a comparative concurrent choice study, shelter dogs stood out as a unique group for their high level of preference for petting (Feuerbacher and Wynne, 2014); they also rapidly formed attachment bonds with a human after only a few social interactions with them (Gácsi et al., 2001) and were shown to remain in proximity with an unknown human for longer than pet dogs (Barrera et al., 2010). It is moreover possible that free-ranging dogs might have a more generalized social response than pet dogs toward humans. Thus, while pet dogs are very friendly with the owners and generally friendly with familiar people, free-ranging dogs may show social behaviors toward a wider range of people, if they have come to view people as a potential source of food and comfort. However, another possibility should be considered, which is that the subjects’ behavior was affected by the test setting. In fact, while free-ranging dogs were tested in their (outdoor) home environment, pet dogs were not. Instead, they were tested in a dog area where dogs might go only for limited periods; thus, they might have been more interested in exploring the environment than staying in proximity to a human. To explore this potential interpretation, we tested an additional group of dogs in a quiet and familiar outdoor environment. The comparison including the two pet dog groups and free-ranging dogs showed that pet dogs tested in the familiar, undisturbed location spent more time being cuddled than pet dogs tested in dog areas, but did not differ from free-ranging dogs. Additionally, they showed a higher likelihood to approach an experimenter in the Test phase than the other two groups. This lends tentative support to the fact that pet dogs tested in dog areas may have been more distracted by their environment. However, it should also be noted that pet dogs tested in dog areas belong to owners that were recruited randomly while walking around with their dogs. Thus, they might be a more representative sample than pet dogs tested in a dog day care facility that might have owners that are more sensitive to dogs’ behavior and thus dogs with a greater ‘socialization’ experiences. Moreover, the environment of the free-ranging dogs also contains more distractor compared to the day care facility despite the fact that it is a familiar environment for the free-ranging dogs. However, these results show that the life experience and context have a huge impact on subjects’ behavior, highly affecting the interpretation of the results. In comparative studies, it should be of primary importance to keep these possible confounding factors into account.

Nevertheless, given that no significant difference between free-ranging dogs and pet dogs tested in dog areas emerged in the likelihood of approaching either one of the two experimenters at the beginning of the Test-phase (69% of free-ranging dogs and 71.7% of pet dogs approached), it would seem that pet dogs were interested in the task as well as free-ranging dogs. Interestingly, almost all pet dogs tested in the day care facility center approached the experimenter (93.5%) and they did it significantly more than pet dogs tested in dog areas and free-ranging dogs. This result could be attributed to them being overall less distracted than pet dogs tested in dog areas as well as free-ranging dogs and/or more attracted to the humans than free-ranging dogs, or, more likely, to the fact that these dogs were used to interact with the trainers of the dog care center. However, we did not find any difference in the frequency of choosing the ‘social experimenter’ over the ‘food experimenter’ (both groups chose 50-50 in the Test phase) for pet dogs tested in dog areas or for free-ranging dogs, as well as for pet dogs tested in dog areas and pet dogs tested in the day care facility. Perhaps the most surprising result is the lack of preference for the ‘food experimenter’ in free-ranging dogs considering the sporadic food availability in their environment. As we already discussed above, subjects might have forgotten which experimenter provided food or social contact and thus approached one or the other human randomly. However, their great interest in approaching and spending time in proximity to the experimenters remains an interesting finding. Free-ranging dogs’ interest in social interactions with humans is in line with recent studies on Indian populations of free-ranging dogs that showed high friendliness toward humans and an understanding of humans’ communicative social cues (Bhattacharjee et al., 2017a, b, 2018,2020).

## Conclusion

In conclusion, our study supports the idea that domestication has affected dogs’ interest in being in proximity to a human partner providing food or petting, and this seems to be the case also in dogs with a relatively sparse socialization experience (free-ranging dogs). However, at present it is not clear what the driving motivation to interact with the human may be, and future studies including group of dogs with different experiences and tested in different contexts as well as physiological measures and more detailed analyses of the types of behaviors exhibited may help answer such questions.

## Data Availability Statement

All datasets generated for this study are included in the article/Supplementary Material.

## Ethics Statement

The animal study was reviewed and approved by the ‘Ethik und Tierschutzkommission’ of the University of Veterinary Medicine of Vienna (Protocol number: ETK-28/07/2017, ETK-05/11/2018). Informed consent was obtained by all owners of the pet dogs. The authorization to test the free-ranging dogs was provided by the municipality of Taghazout (Morocco). Written informed consent was obtained from the owners for the participation of their animals in this study.

## Author Contributions

ML, FR, and SM-P designed the study. ML, JB, KP, and KS prepared the study material, data acquisition, entered the data, and prepared it for statistical analyses. ML analyzed the data and wrote the first draft of the manuscript. All authors interpreted the data, contributed to manuscript revision, read and approved the submitted version.

## Conflict of Interest

The authors declare that the research was conducted in the absence of any commercial or financial relationships that could be construed as a potential conflict of interest.

## Footnotes

1 www.wolfscience.at
