# Supplementary material for: The Effect of Domestication and Experience on the Social Interaction of Dogs and Wolves With a Human Companion
Source: Front Psychol. 2020 Apr 23;11:785. doi: 10.3389/fpsyg.2020.00785 (PMC7197371; doi:10.3389/fpsyg.2020.00785)
Supplement: Supplementary file 1 [file Table_1.DOCX]

Supplementary Material

# Supplementary Table

**Table 1. Sample sizes per model and number of negative and positive responses in the case of logistic models.**

| Data set | Response | Error distribution; link function | nr. obs. | nr. indiv. | nr. no | nr. yes |
| --- | --- | --- | --- | --- | --- | --- |
| WSCd vs. WSCw | Proportion time in contact with CP in Pre-test | Beta; logit | 58 | 29 | NA | NA |
|  | Approaches any human | Binomial; logit | 58 | 29 | 19 | 39 |
|  | Approaches CP | Binomial; logit | 39 | 25 | 21 | 18 |
|  | Proportion time in proximity of CP | Beta; logit | 41 | 25 | NA | NA |
| FRd vs. PdA | Proportion time in contact CP in the Pre-test | Beta; logit | 98 | 98 | NA | NA |
|  | Approaches any human | Binomial; logit | 98 | 98 | 29 | 69 |
|  | Approaches CP | Binomial; logit | 69 | 69 | 40 | 29 |
|  | Proportion time in proximity of CP | Beta; logit | 61 | 61 | NA | NA |
| PdA vs. PdC vs. PdA | Proportion time in contact CP in the Pre-test | Beta; logit | 129 | 129 | NA | NA |
|  | Approaches any human | Binomial; logit | 129 | 129 | 31 | 98 |
|  | Approaches CP | Binomial; logit | 98 | 98 | 54 | 44 |
|  | Proportion time in proximity of CP | Beta; logit | 73 | 73 | NA | NA |
